# Host Genetic Risk Factors for West Nile Virus Infection and Disease Progression

**DOI:** 10.1371/journal.pone.0024745

**Published:** 2011-09-15

**Authors:** Abigail W. Bigham, Kati J. Buckingham, Sofia Husain, Mary J. Emond, Kathryn M. Bofferding, Heidi Gildersleeve, Ann Rutherford, Natalia M. Astakhova, Andrey A. Perelygin, Michael P. Busch, Kristy O. Murray, James J. Sejvar, Sharone Green, John Kriesel, Margo A. Brinton, Michael Bamshad

**Affiliations:** 1 Department of Pediatrics, University of Washington, Seattle, Washington, United States of America; 2 Department of Biostatistics, University of Washington, Seattle, Washington, United States of America; 3 Department of Internal Medicine, Division of Infectious Diseases, University of Utah, Salt Lake City, Utah, United States of America; 4 Department of Biology, Georgia State University, Atlanta, Georgia, United States of America; 5 Blood Systems, San Francisco, California, United States of America; 6 School of Public Health, University of Texas Health Sciences Center at Houston, Houston, Texas, United States of America; 7 National Center for Emerging and Zoonotic Infectious Diseases, Centers for Disease Control and Prevention, Fort Collins, Colorado, United States of America; 8 Department of Medicine, Center for Infectious Disease and Vaccine Research, University of Massachusetts Medical School, Worcester, Massachusetts, United States of America; Food and Drug Administration, United States of America

## Abstract

West Nile virus (WNV), a category B pathogen endemic in parts of Africa, Asia and Europe, emerged in North America in 1999, and spread rapidly across the continental U.S. Outcomes of infection with WNV range from asymptomatic to severe neuroinvasive disease manifested as encephalitis, paralysis, and/or death. Neuroinvasive WNV disease occurs in less than one percent of cases, and although host genetic factors are thought to influence risk for symptomatic disease, the identity of these factors remains largely unknown. We tested 360 common haplotype tagging and/or functional SNPs in 86 genes that encode key regulators of immune function in 753 individuals infected with WNV including: 422 symptomatic WNV cases and 331 cases with asymptomatic infections. After applying a Bonferroni correction for multiple tests and controlling for population stratification, SNPs in *IRF3* (OR 0.54, p = 0.035) and *MX1*, (OR 0.19, p = 0.014) were associated with symptomatic WNV infection and a single SNP in *OAS1* (OR 9.79, p = 0.003) was associated with increased risk for West Nile encephalitis and paralysis (WNE/P). Together, these results suggest that genetic variation in the interferon response pathway is associated with both risk for symptomatic WNV infection and WNV disease progression.

## Introduction

Despite vast improvements in medicine and health care, humans still suffer unpredictably from epidemics of infectious disease. One such pathogen for which infection results in wide variation in clinical presentations and outcomes is West Nile virus (WNV). WNV is a neurotropic, mosquito-borne RNA virus that belongs to the Japanese encephalitis virus serocomplex in the family *Flaviviridae*, and is closely related to viruses that cause dengue fever/hemorrhagic fever, yellow fever, and Japanese encephalitis. The primary transmission cycle of WNV occurs between mosquitoes (primarily *Culex* species), the insect vector, and birds, the reservoir host. Incidental infections occur when mosquitoes transmit WNV to mammalian “dead-end” hosts including humans and horses.

Human WNV was first isolated from a febrile woman in the West Nile province of Uganda in 1937 [1]. Subsequent outbreaks have been reported in Africa, the Middle East, Western Asia, and Europe [2], [3], [4]. In 1999, WNV emerged in North America and spread from New York City across the continental US and into Canada, Central, and South America [5], [6], [7]. As of December 31, 2010, the number of documented WNV disease cases and deaths reported to the Centers for Disease Control and Prevention (CDC) totaled 30,584 and 1,214, respectively (http:///www.cdc.gov). Accordingly, WNV poses an increasing threat to public health, making it important to identify factors that predispose humans to WNV-induced disease and death.

The clinical course of individuals with WNV infection can be divided into three categories: asymptomatic infections, uncomplicated West Nile fever (WNF), and West Nile neuroinvasive disease (WNND). Conventionally, WNF is defined as an acute systemic febrile illness in the absence of neurological signs, whereas WNND is characterized by meningitis, encephalitis, paralysis, and/or death. WNND is rare among WNV-infected individuals with <1% of the WNV infected individuals estimated to develop central nervous system infection [8]. Increased risk of neuroinvasive disease is reported among immunocompromised individuals and the elderly. However, WNND has been reported among healthy young individuals, indicating that clinical outcome is not solely affected by immune senescence or suppression [9].

Identifying the genetic factors (i.e. risk alleles) that influence the development of WNV disease could help to elucidate pathways important for increased pathogenicity of WNV disease, facilitate the identification of individuals at high risk for severe WNV-induced disease, and provide potential therapeutic targets. However, to date, few such alleles have been identified [10], [11], [12], [13]. A 32-bp deletion in the coding region of the CC chemokine receptor 5 (*CCR5Δ32*) was previously reported to be associated with both increased susceptibility to WNV infection and death [12], [13]. Also, allelic variants in two of the genes that encode the antiviral enzyme 2′–5′ oligoadenylate synthetase (OAS), *OAS1* and *OASL*, have been associated with WNV susceptibility or WNND, although subsequent attempts to replicate the association with *OASL* were unsuccessful [10], [11].

We undertook a case-control association study of candidate genes to search for loci influencing susceptibility and resistance to WNV infection and disease progression. Significant associations between WNV infection and SNPs in *IRF3*, *MX1*, and *OAS1* were found. No significant associations were identified between WNV disease and *CCR5Δ32* or polymorphisms in the 5′ *cis*-regulatory region of *CCR5*.

## Results

### Study Participants

To test for SNP associations with WNV infection, symptomatic WNV-positive patients (n = 422) were compared to asymptomatic (n = 331) controls. To test for SNP associations with disease progression, we categorized symptomatic WNV-positive cases as either West Nile fever/meningitis (WNF/M) (n = 280) or West Nile encephalitis/paralysis (WNE/P) (n = 140) cases. We chose not to use the conventional distinction of WNF and WNND for two reasons. First, the clinical course of infection for WNV patients presenting with meningitis is more similar to that for WNF patients than it is to patients who develop encephalitis or paralysis. Second, a fraction of the patients with meningitis were never administered a lumbar tap to confirm meningitis. Thus, patients with meningitis could have been included in the WNF or WNND categories. For the three significant associations identified as part of this study, an additional matched control group of 300 random blood donors (RBD) collected in the United States with unknown exposure history to WNV was included in the analysis referred to herein as RBD. The RBDs were included in the analysis in order to compare our results to previously published results wherein similar controls were used. Demographic characteristics for each case category are summarized in Table 1.

**Table 1:** Subject characteristics*.

Characteristic	WNE/P (n = 140)	WNF/M (n = 280)	Asymptomatic (n = 331)	RBD (n = 300)
Median Age (y)	57	50	47	8
% of samples with age data	93.62	96.42	29.61	100
Female (%)	43.26	59.86	41.99	52
African American (%)^a^	3.57	2.5	0	0.33
East Asian (%)^a^	0.71	0	0.3	0
European (%)^a^	71.43	80	27.79	99
Hispanic (%)^a^	4.29	1.43	0.6	0
Native American (%)^a^	0.71	0	0.3	0.33
Other (%)^a^	1.43	2.5	0.3	0.33
Unknown (%)^a^	17.86	13.57	70.69	0
Fever (%)^b^	43.26	67.38	NA	NA
Meningitis (%)^b^	54.61	25.81	NA	NA
Encephalitis (%)^b^	80.85	0	NA	NA
Paralysis (%)^b^	31.91	0	NA	NA
Mortality (%)^b^	0	0	NA	NA

Subjects were classified into four disease phenotypes including West Nile encephalitis/paralysis (WNE/P), West Nile fever/meningitis (WNF/M), asymptomatic disease, and random blood donors (RBD). The RBD controls were only genotyped for the 3 significant SNPs.

*The two study subjects for whom disease severity is unknown are not presented. *CCR5* genotyping was performed on a subset of the samples presented here. Please refer to the Methods for a description of the *CCR5* sample sizes in each WNV category.

a self-reported ancestry.

b clinical outcome categories are not mutually exclusive.

NA, not applicable.

### Selection of candidate genes and SNPs

We selected 57 candidate genes for association testing on the risk of WNV disease in our cohort based on several criteria including: (1) previously reported association with risk of WNV disease in humans; (2) data supporting involvement in protection from WNV pathogenesis in an animal model; (3) direct influence on WNV replication efficiency demonstrated in an *in vitro* model. Additional candidates included 29 genes encoding host proteins involved in the adaptive or innate immune responses that were either known or predicted to interact with host proteins involved in WNV infection [14]. In an effort to discover SNPs in these 29 genes, we sequenced either the entire gene (i.e., exons and introns in genes <10 kb in length) or only the protein-coding exons (for genes >10 kb in length) as well as putative regulatory regions within 1.5 kb upstream of the transcription initiation site in 93 unrelated individuals from three continental groups of the Human Genome Diversity Project-Centre d'Etude Polymorphisme Humain (HGDP-CEPH). These individuals included 31 sub-Saharan Africans (Bantu, Mandenka), 31 Europeans (Bergamo, Russian, French, Orcadian), and 31 East Asians (Cambodian, Dai, Daur, Han, Hezhen, Japanese, Lahu, Maiozu).

For each candidate gene, haplotypes were inferred for each continental population and SNPs that tagged each haplotype with a frequency >5% in Europeans were selected to be genotyped in the entire cohort. Previously described functional SNPs or newly identified SNPs that were predicted to have functional consequences were also selected for genotyping. In total, 384 SNPs were genotyped using an Illumina custom oligo pool assay (OPA) (Table S1). In addition, we sequenced the 5′ *cis*-regulatory region of *CCR5* and genotyped the *CCR5Δ32* deletion in each individual

### Analysis of symptomatic WNV infection

**Figure 1:**
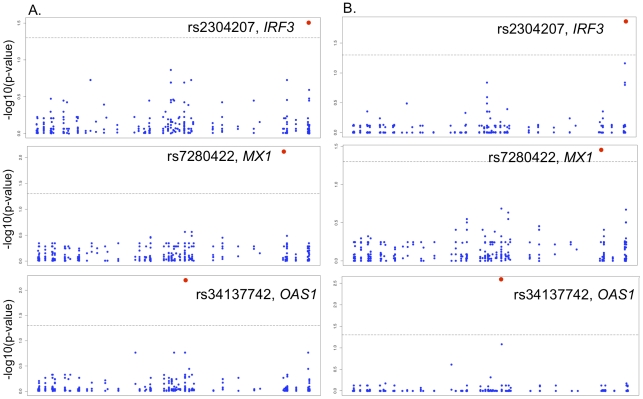
Significant SNPs associated with symptomatic WNV infection or West Nile encephalitis/paralysis (WNE/P). Three SNPs were significantly associated with symptomatic WNV infection and WNE/P. These include rs2304207 in *IRF3* for symptomatic cases vs. asymptomatic controls for a dominant inheritance model, rs7280422 in *MX1* for symptomatic cases vs. asymptomatic controls for a recessive inheritance model and rs34137742 in *OAS1* for WNE/P vs. West Nile fever/meningitis (WNF/M) using a recessive inheritance model. Contingency table results are presented in A whereas results for the logistic regression are shown in B. The horizontal axis for each plot shows the genomic position arranged in order of chromosomal position for each SNP in the dataset. The vertical axis depicts the negative log p-values generated from the chi-square distribution or the logistic regression model analysis. All p-values were corrected for multiple tests using the Bonferroni correction. The black dashed line indicates the five percent significance threshold corrected for multiple tests. No significant SNP associations were identified for WNE/P versus WNF/M for a dominant inheritance model.

To test for SNPs associated with symptomatic WNV infection, all WNV-positive cases (i.e., WNF/M and WNE/P cases) were compared with asymptomatic, WNV-positive blood donors (Tables S2–S8). A genotypic contingency analysis (3×2) revealed a statistically significant association between rs2304207 in intron 2 of *IRF3* (p = 0.0399) and symptomatic WNV disease (Table S2). None of the other associations reached statistical significance. We also considered dominant, recessive, and additive genetic models (Tables S3–S5). Under a dominant model, *IRF3* rs2304207 was significantly associated with symptomatic WNV infection (OR = 0.52, p = 0.007) (Table 2 and Figure 1A). The GC and GG genotypes were more frequent in asymptomatic WNV-positive controls than in symptomatic cases. Using a recessive model, *MX1* rs7280422 was significantly associated with symptomatic infection (OR = 0.25, p = 0.042) (Table 2 and Figure 1A). Specifically, the reference homozygote genotype, CC, was more common in asymptomatic WNV-positive controls than in symptomatic cases.

**Table 2:** Contingency table analysis odds ratios (OR) of significant SNPs associated with WNV symptomatic infection or West Nile encephalitis/paralysis (WNE/P).

Disease Model	Inheritance Model	SNP	Gene	Symptomatic vs. Asymptomatic	Symptomatic vs. RBD
Symptomatic	Dominant	rs2304207	*IRF3*	0.52**	0.97
Infection		rs7280422	*MX1*	0.74	0.84
		rs34137742	*OAS1*	0.77	0.88
	Recessive	rs2304207	*IRF3*	0.72	1.32
		rs7280422	*MX1*	0.25*	0.80
		rs34137742	*OAS1*	1.33	1.23

				WNE/P vs. WNF/M+Asymptomatic	WNE/P vs. RBD	WNE/P vs. WNF/M+RBD	WNE/P vs. WNF/M+Asymptomatic+RBD
WNE/P	Dominant	rs2304207	*IRF3*	0.70	1.03	1.00	0.78
		rs7280422	*MX1*	1.22	1.14	1.33	1.20
		rs34137742	*OAS1*	0.96	0.97	1.04	0.96
	Recessive	rs2304207	*IRF3*	0.92	1.45	1.29	1.05
		rs7280422	*MX1*	0.38	0.81	0.89	0.47
		rs34137742	*OAS1*	5.61**	3.49**	5.38****	4.67****

Odds ratios (ORs) are reported for the minor allele.

a Two disease models were used and include WNV symptomatic infection and West Nile encephalitis/paralysis (WNE/P). To identify associations with symptomatic infection in our cohort, symptomatic WNV was compared to asymptomatic infection. To identify associations with WNE/P in our cohort, we compared WNE/P to West Nile fever/meningitis (WNF/M) combined with asymptomatic infection. To understand if our associations were robust to alterations in the control group definition, we compared symptomatic infection vs. random blood donors (RBD), WNE/P vs. RBD, WNE/P vs. WNF/M combined with RBD, and WNE/P vs. WNF/M+Asymptomatic+RBD.

*p<0.05,

**p<0.01,

***p<0.001,

****p<0.0001.

P-values were corrected for multiple tests using the Bonferroni Correction.

Logistic regression, controlled for population stratification, was performed for each SNP separately and recapitulated the results of the contingency table analysis (Tables S6–S8). *IRF3* rs2304207 was significantly associated with symptomatic WNV disease (OR = 0.54 [95% confidence interval (CI) 0.39–0.74]; p = 0.035) under a dominant genotypic model (Table 3) with the GC and GG genotypes found significantly more often in asymptomatic WNV-positive controls than in WNV symptomatic cases. Under a recessive model, *MX1* rs7280422 was associated with symptomatic infection (OR = 0.19 [95% CI 0.08–0.42], p = 0.014) with the homozygous reference allele CC found significantly more often in asymptomatic WNV-positive controls than in WNV symptomatically infected individuals (Table 3 and Figure 1B). No significant association with WNV infection was identified using an additive model (Table S6).

**Table 3:** Logistic regression analysis odds ratios (OR) of significant SNP associations with WNV infection or West Nile encephalitis/paralysis (WNE/P).

Disease Model^a^	Inheritance Model	SNP	Gene	Chr	Function	Alleles	Reference Allele	Rank^b^	OR^c^	p*
Symptomatic	Dominant	rs2304207	*IRF3*	19	Intron 2	G/C	G	1	0.54	**0.035**
Infection		rs7280422	*MX1*	21	Intron 3	C/G	C	10	0.65	0.313
		rs34137742	*OAS1*	12	Intron 2	C/T	C	301	0.97	0.996
	Recessive	rs7280422	*MX1*	21	Intron 3	C/G	C	1	0.19	**0.014**
		rs34137742	*OAS1*	12	Intron 2	C/T	C	67	1.98	0.769
		rs2304207	*IRF3*	19	Intron 2	G/C	G	151	0.72	0.984
WNE/P	Dominant	rs2304207	*IRF3*	19	Intron 2	G/C	G	54	0.71	0.712
		rs34137742	*OAS1*	12	Intron 2	C/T	C	166	1.24	0.901
		rs7280422	*MX1*	21	Intron 3	C/G	C	322	0.99	0.963
	Recessive	rs34137742	*OAS1*	12	Intron 2	C/T	C	1	9.79	**0.003**
		rs7280422	*MX1*	21	Intron 3	C/G	C	15	3.73	0.750
		rs2304207	*IRF3*	19	Intron 2	G/C	G	284	1.04	1.000

OR, odds ratio; chr, chromosome.

ORs are reported for the minor allele.

a Two disease models were used and include WNV symptomatic infection vs. asymptomatic infection (symptomatic infection) and WNE/P vs. WNF/M combined with asymptomatic infection (WNE/P).

b The p-value rank of the 360 SNPs tests.

c Results were obtained using logistic regression that controlled for population stratification using the PC method.

*p-values were corrected for multiple tests using the Bonferroni correction.

Asymptomatic WNV-positive individuals were used as controls rather than individuals with unknown WNV exposure history because we thought the former enabled a more powerful test (i.e., it is more likely that individuals with unknown exposure history have never been exposed to WNV than it is that some of the putatively asymptomatic WNV-positive cases really were symptomatic) [14]. Nevertheless, 300 RBD with unknown exposure history to WNV were genotyped for rs2304207 and rs7280422 and the data compared to that for all symptomatic WNV cases (i.e., WNF/M and WNE/P). We included this additional control group so that our results would be directly comparable to studies conducted previously. No significant associations were detected for *IRF3* (dominant: OR = 0.97 [95% CI 0.69–1.35] p = 0.836) or *MX1* (recessive: OR = 0.80 [95% CI 0.31–2.11] p = 0.655) SNPs (Table 2).

### Analysis of WNV Disease Progression

To identify SNP associations with WNV disease progression (defined here as the presence of WNE/P), WNE/P cases were compared to a control group consisting of WNF/M individuals plus asymptomatic, WNV-positive blood donors. A genotypic contingency analysis (3×2) revealed a statistically significant association between rs34137742 in *OAS1* (p = 0.012) and risk of WNE/P (Table S9). In addition, dominant, recessive, and additive genetic models were considered (Tables S10–S12). Under a recessive model, the homozygote non-reference genotype, TT, of *OAS1* rs34137742 was significantly associated with WNE/P (OR = 5.61, p = 0.006) (Table 2 and Figure 1A). Using logistic regression, this result was recapitulated under a recessive genotypic model (OR = 9.79, [95% CI 3.60–26.61], p = 0.003) (Table 3, Figure 1B, and Table S15). No significant associations with WNE/P were identified using a dominant or an additive model of inheritance (Tables S13 and S14).

**Figure 2:**
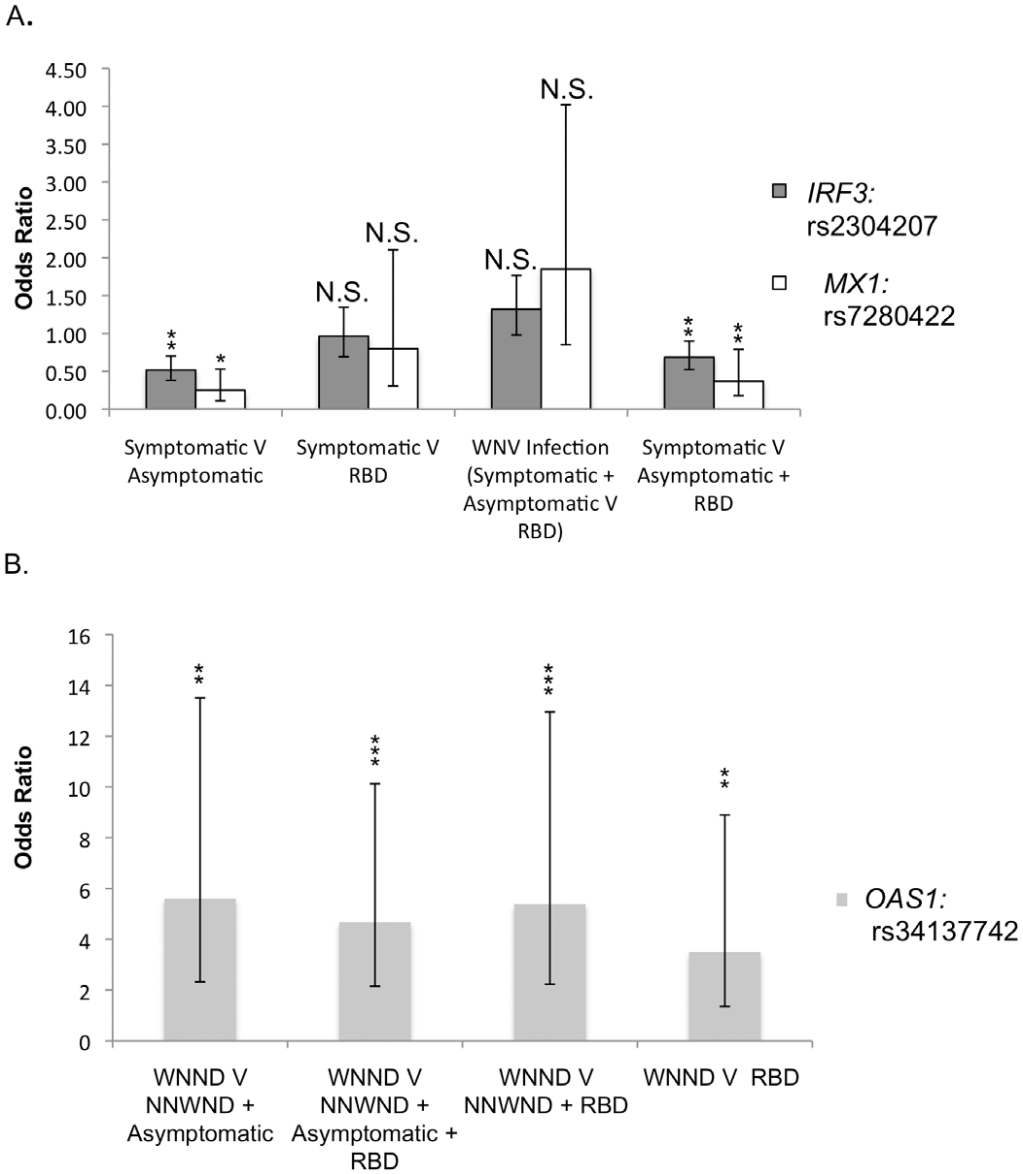
Significant SNP associations depend on control group for symptomatic infection. Minor allele odds ratios (ORs) (bars) were calculated using a 2×2 contingency table analysis. The 95% confidence intervals (CIs) (lines) were calculated for each significant association. Statistical significance is shown above each bar where * p<0.05, **p<0.01, ***p<0.001, and N.S. is non-significant. A. *IRF3* SNP rs2304207 and *MX1* SNP rs7280422 were tested for associations with symptomatic WNV infection. B. *OAS1* SNP rs34137742 was tested for an association with WNE/P. ORs for a recessive model of inheritance were plotted for *MX1* and *OAS1*. ORs for a dominant model of inheritance were plotted for *IRF3*. The results of the WNE/P analysis were robust to alterations in the definition of the control group, but the results of the symptomatic infection analysis were not. This suggests that selection of control group affects the SNP associations with symptomatic WNV infection.

The association between *OAS1* rs34137742 and WNE/P was robust to alterations in the definition of the control group as shown by comparing the following three donor groups: 1) WNE/P vs. RBD 2) WNE/P vs. WNF/M and RBD and 3) WNE/P vs. WNF/M, asymptomatic controls, and RBD. Significant associations were detected between WNE/P vs. RBD (OR = 3.49 [95% CI 1.36–8.90] p = 0.0057), WNE/P vs. WNF/M and RBD (OR = 5.38 [95% CI 2.23–12.95] p = 0.000033), and WNE/P vs. WNF/M, asymptomatic controls, and RBD combined (OR = 4.67 [95% CI 2.15–10.13] p = 0.00002) (Table 2; Figure 2b). In each comparison, the TT genotype of *OAS1* rs34137742 was significantly associated with WNE/P (Table 2). We also tested if the significant association we detected for this SNP and WNE/P was identified when using the conventional WNV phenotype classification. For this analysis, we compared WNND cases to a control group composed of individuals with WNF and asymptomatic, WNV-positive blood donors. All WNV patients reporting meningitis were included in the WNND group. We did not detect an association between *OAS1* rs34137742 and WNND using a dominant (logistic regression OR = 0.83 [95% CI 0.58–1.21], p = 114.78) or a recessive (logistic regression OR = 3.66 [95% CI 1.52–8.84], p = 1.29) model of inheritance after correcting for multiple tests.

### Analysis of variants previously associated with WNV disease

SNPs in *OAS1* rs10774671 and *OASL* rs3213545 previously have been associated with increased risk of WNV infection (i.e., WNV-positive status) and symptomatic WNV infection, respectively [10], [11]. In our cohort, we found no evidence of a significant association with symptomatic infection for *OAS1* SNP rs10774671 using a dominant model (OR 1.21, p = 65.31), or a recessive model (OR 1.30, p = 81.66). Likewise, no significant associations were detected for *OASL* SNP rs3213545 using a dominant model (OR 0.78, p = 34.00) or recessive model (OR 0.52, p = 9.32) (Table 4). Results of the logistic regression were consistent with the results of the contingency table analysis (Tables S7, S8). Additionally, neither SNP was associated with WNE/P (Table 4 and Tables S9–S15).

**Table 4:** Contingency table analysis odds ratios of SNPs previously associated with WNV infection, symptomatic infection, or disease progression.

Disease Model^a^	Inheritance Model	SNP	Gene	Symptomatic vs. Asymptomatic	Symptomatic vs. WNV-Neg^b^
Symptomatic	Dominant	*CCR5Δ32*	*CCR5*	2.14	1.38*
Infection		rs10774671	*OAS1*	1.21	0.97
		rs3213545	*OASL*	0.78	0.72*
	Recessive	*CCR5Δ32*	*CCR5*	1.03	1.32
		rs10774671	*OAS1*	1.30	0.89
		rs3213545	*OASL*	0.52	0.45**

				WNE/P vs. WNF/M+Asymptomatic	WNE/P vs. WNV-Neg^b^
WNE/P^c^	Dominant	*CCR5Δ32*	*CCR5*	0.74	1.45
		rs10774671	*OAS1*	1.20	1.03
		rs3213545	*OASL*	0.64	0.56**
	Recessive	*CCR5Δ32*	*CCR5*	1.08	0.77
		rs10774671	*OAS1*	1.77	1.25
		rs3213545	*OASL*	0.27	0.20**

				WNV-Pos^d^ vs. WNV-Neg^d^
WNV Infection	Dominant	*CCR5Δ32*	*CCR5*	1.36**
		rs10774671	*OAS1*	0.89
		rs3213545	*OASL*	0.80
	Recessive	*CCR5Δ32*	*CCR5*	1.00
		rs10774671	*OAS1*	0.79
		rs3213545	*OASL*	0.63*

Odds ratios (ORs) are reported for the minor allele.

a Three disease models were used and include WNV symptomatic infection vs. asymptomatic infection (symptomatic infection), WNE/P vs. WNF/M combined with asymptomatic infection (WNE/P), and WNV infection vs. WNV-negative (WNV Infection).

b WNV-negative controls (WNV-Neg). For *OAS1* and *OASL*, the Lim *et al.* WNV-negative samples were used as a control group [11]. For *CCR5*, the Glass et al. WNV sero-negative cohort was used as a control group [13].

c West Nile encephalitis/paralysis (WNE/P).

d WNV-positive cases (WNV-Pos) consisted of WNV symptomatic and asymptomatic WNV-positive cases.

*p<0.05,

**p<0.01,

***p<0.001,

****p<0.0001.

P-values were corrected for multiple tests using the Bonferroni Correction.

Next, we attempted to replicate the previously identified associations with WNV infection in our cohort by comparing all WNV-positive cases (WNE/P, WNF/M, and asymptomatic cases) (n = 751 *OAS1* rs10774671 or n = 749 *OASL* rs3213545) to the WNV-negative controls from Lim *et al.* (n = 552) [11]. The WNV-negative controls used by Lim *et al.* consisted of random blood donors collected prior to the 1999 introduction of WNV to the United States (n = 360) and U.S. blood donors collected by the American Red Cross who were identified as WNV false positives (n = 192). *OAS1* rs10774671 was not associated with risk of infection under a dominant (OR = 0.89 [95% CI 0.71–1.11], p = 0.301) or a recessive (OR = 0.79 [95% CI 0.58–1.09], p = 0.150) model. In contrast, *OASL* rs3213545 was significantly associated with WNV infection under a recessive (OR = 0.63 [95% CI 0.42–1.58], p = 0.025) model of inheritance (Table 4). Interestingly, Lim *et al.* did not find an association between *OASL* rs3213545 and WNV infection, even though we did find an association. Thus, we could replicate neither of the results obtained by Lim *et al.* [11].

We repeated the tests of association between *OAS1* rs10774671 and *OASL* rs3213545 and individuals with symptomatic WNV infection (WNE/P and WNF/M) or WNE/P using the Lim *et al.* WNV-negative controls for comparison [11]. Once again, contingency table analysis revealed no association for symptomatic infection and *OAS1* rs10774671 genotype using a dominant (OR = 0.97 [95% CI 0.75–1.26], p = 0.81) or a recessive model (OR = 0.89 [95% CI 0.62–1.27], p = 0.51) (Table 4). Likewise, no association for WNE/P and *OAS1* rs10774671 was detected (dominant: OR = 1.03 [95% CI 0.6–1.50], p = 0.891, recessive: OR = 1.25 [95% CI 0.77–2.02], p = 0.372) (Table 4). *OASL* rs3213545 was significantly associated with both symptomatic infection (dominant: OR = 0.72 [95% CI 0.56–0.93], p = 0.012, recessive: OR = 0.45 [95% CI 0.27–0.77], p = 0.003) and WNE/P (dominant: OR = 0.56 [95% CI 0.38–0.81], p = 0.002, recessive: OR = 0.20 [95% CI 0.06–0.65], p = 0.003) (Table 4).

*CCR5Δ32* has been associated previously with an increased risk of symptomatic WNV infection [12], [13], [15]. We tested whether *CCR5Δ32* was associated with risk of symptomatic WNV infection or disease progression in our cohort of WNE/P, WNF/M, and asymptomatic WNV-positive cases. No significant associations were detected for symptomatic WNV infection or WNE/P, under either a dominant or recessive model of inheritance, by contingency analysis or logistic regression analysis (Table 4 and Table S16). Next, to test whether *CCR5Δ32* was associated with risk of WNV infection, we compared all WNV-positive cases (i.e. WNE/P, WNF/M, and asymptomatic cases) in our cohort (n = 713) to published genotype data from the Glass *et al.* WNV sero-negative cohort presenting with acute illness, but in whom WNV had been ruled out by serological testing (n = 1,318) [13]. A significant association was identified for risk of WNV infection under a dominant model (OR = 1.36 [95% CI 1.08–1.71], p = 0.009) (Table 4). We also tested for risk of symptomatic WNV infection and WNE/P using the Glass *et al.* WNV sero-negative controls rather than WNV-positive asymptomatic blood donors [13]. *CCR5Δ32* was significantly associated with symptomatic infection under a dominant model of inheritance (OR = 1.38 [95% CI 1.04–1.83], p = 0.026) (Table 4 and Table S17). No association was identified with WNE/P using a dominant (OR = 1.45 [95% CI 0.94–2.25], p = 0.094) or recessive (OR = 0.77 [95% CI 0.10–5.95], p = 0.803) model (Table 4 and S17).

SNPs in the 5′ *cis*-regulatory region of *CCR5* have been associated with CCR5 mRNA expression levels, risk of mother-to-child HIV-1 transmission, and disease progression of HIV-1 infected children and adults [16], [17], [18]. Given the influence of *CCR5* regulatory variants on HIV-1 disease as well as the reported association between *CCR5Δ32* and WNV infection, we hypothesized that variants in the 5′ *cis*-regulatory region of *CCR5* also may be associated with WNV infection or disease progression. We looked for associations between SNPs in this region and symptomatic infection and WNE/P using our cohort of WNE/P (n = 134), WNF/M (n = 258), and asymptomatic controls (n = 330). However, none of the common SNPs in 5′ *cis*-regulatory region of *CCR5* were significantly associated with WNE/P or WNV infection using data from our cohort (data not shown).

## Discussion

From a set of 86 candidate genes, we identified variants in three loci (*IRF3*, *MX1*, and *OAS1*) that were associated with WNV disease (Table 5). For two of these loci, *IRF3* and *MX1*, no association with WNV disease has been reported previously in humans. *OAS1* and one of its murine orthologues, *Oas1b*, have been associated with modulating WNV disease in humans and mice, respectively [11], [19], [20,].

**Table 5:** Summary of significant results.

Inheritance Model	Gene	SNP	Cases (n)	Controls (n)	Symptomatic Infection	WNE/P^a^	WNV Infection
Dominant	*CCR5*	*CCR5Δ32*	WNV-Pos^b^ (713)	WNV-Neg^c^ (1318)			X
			symptomatic (385)	WNV-Neg^c^ (1318)	X		
	*IRF3*	rs2304207	symptomatic (419)	asymptomatic (331)	X		
	*OASL*	rs3213545	symptomatic (419)	WNV-Neg^c^ (552)	X		
			WNE/P^a^ (139)	WNV-Neg^c^ (552)		X	
Recessive	*MX1*	rs7280422	symptomatic (418)	asymptomatic (329)	X		
	*OAS1*	rs34137742	WNE/P^a^ (126)	WNF/M^d^+asymptomatic (596)		X	
	*OASL*	rs3213545	Symptomatic (419)	WNV-Neg^c^ (552)	X		
			WNE/P^a^ (139)	WNV-Neg^c^ (552)		X	
			WNV-Pos^b^ (749)	WNV-Neg^c^ (552)			X

Significant SNP associations with WNV infection, symptomatic infection, or WNE/P are reported for the significant SNPs identified herein and those previously shown to have an association. For those SNPs previously associated with West Nile disease, significant results were obtained by performing an analysis using our data combined with published data. An X indicates that a significant association (p<0.05) was identified for the corresponding SNP using the case/control groups as indicated.

Sample sizes may vary from the total number reported in Table 1 as a result of missing genotypes for a particular SNP.

a West Nile encephalitis/paralysis (WNE/P).

b WNV-positive cases (WNV-Pos) consisted of WNV symptomatic and asymptomatic WNV-positive cases.

c WNV-negative controls (WNV-Neg). For *OASL*, the Lim *et al.* WNV-negative samples were used as a control group [11]. For *CCR5*, the Glass *et al.* WNV sero-negative cohort was used as a control group [13].

d West Nile fever/meningitis (WNF/M).

*IRF3* encodes a member of the interferon regulatory transcription factor family involved in the upregulation of type 1 IFN genes as well as other pathway genes. However, IRF-3 has been reported to protect mice from WNV-induced disease by both interferon-dependent and independent mechanisms [21], [22]. After a peripheral WNV infection, *Irf3^−/−^* mice exhibited increased mortality, earlier viral entry into the CNS, and increased virus levels in the brain and spinal cord compared to wild-type mice [22]. *Irf3^−/−^* mice also exhibit enhanced WNV infection in macrophages *in vivo* and *ex vivo* [22]. Increased replication of WNV in macrophages in the periphery would be expected to enhance disease symptoms consistent with the observed association of *IRF3* variation with human symptomatic WNV infections. Accordingly, *IRF3* is a compelling candidate for influencing the risk of symptomatic WNV infection in humans.

*MX1* belongs to the MX (myxovirus resistance) family of interferon-induced proteins that are GTPases with antiviral functions [23]. Upon viral infection, a host cell secretes type 1 interferons that, in turn, induce the production of MX proteins that diminish viral replication. In mice, *Mx1* confers resistance to orthomyxoviruses including influenza viruses, but has not been demonstrated to confer resistance to flaviviruses [24]. However, it is possible that *MX1* may have an effect on flavivirus infections in humans.

The products of *OAS1*, *OAS2*, *OAS3*, *OASL*, and their downstream effector *RNASEL* each influence host defense by blocking viral replication [23]. Evidence from human cell culture indicates that OAS gene products have an antiviral effect on flavivirus infections [25]. The products of the *Oas1b* alleles differentially affect susceptibility to flavivirus-induced disease in mice, but by an RNase L independent mechanism [19], [20]. In the present study, a single variant in *OAS1*, SNP rs34137742 located in intron 2, was identified as a risk factor for human WNV disease progression, although no association with symptomatic infection as a whole was identified.

*OAS1* rs10774671 was previously reported to be associated with risk of WNV infection [11]; however, we were unable to replicate this finding using our asymptomatic controls or the control samples of Lim *et al.* [11]. In contrast, an association between another SNP, *OAS1* rs34137742, and risk for WNE/P was found using multiple, different control populations (1. WNF/M plus asymptomatic WNV-positive cases, 2. RBD, 3. WNF/M plus RBD, and 4. WNF/M plus asymptomatic WNV-positive cases and RBD). Furthermore, we found an association between *OASL* rs3213545 and risk for WNE/P, symptomatic disease, and infection, but only with the control samples of Lim *et al.* [11]. The observation that *OASL* is associated with WNV disease confirms a previously reported association discovered in a very small sample of cases (n = 27) that failed replication in a second study using the same control samples as included here, but different WNV-positive samples [10], [11].

We did not use the conventional phenotypic classification method of WNND and WNF. Instead, we grouped West Nile patients presenting with fever and/or meningitis into a single category, WNF/M, and categorized patients presenting with encephalitis and/or paralysis as WNE/P. Our decision was based on two observations. First, WNV patients presenting with meningitis are clinically more similar to WNF patients than they are to WNE/P patients. Second, patients classified as WNF are likely to include individuals with unconfirmed meningitis [26]. Therefore, patients with meningitis may be included in both the WNF and WNND categories. When removing West Nile meningitis patients from the analysis, the association of *OAS1* rs34137742 with WNE/P remained significant, but our power to detect this association decreased. However, if WNND patients were compared to WNF plus asymptomatic controls, no association with *OAS1* rs34137742 and WNND was detected (Table S20). These results illustrate the importance of accurate phenotypic classification, as the associations are contingent upon the classification system. The difference in our phenotypic classification of WNV-positive samples compared to that used by Lim *et al.* could explain why we detected an association between *OASL* rs3213545 and risk for WNE/P whereas the Lim *et al.* study did not detect an association between this SNP and WNND [11]. However, this difference in the classification system cannot explain our inability to replicate the association for *OAS1* rs10774671 and WNV infection, as this analysis compared all WNV-positive cases to WNV-negative controls.

It has been shown that the host response to viral infections does not depend on host factors alone. Rather, the virulence of the infecting virus strain may impact the clinical course of infection and could therefore influence the likelihood of disease progression from asymptomatic infection to more severe forms of a disease. For example, particular strains of Dengue virus have been shown to be more virulent than others [27]. Analysis of the specific WNV viral strains infecting each of the participants in this study was beyond the scope of this research. However, it is important to point out that varying degrees of virulence between infecting strains may have impacted WNV pathogenesis in our cohort. Furthermore, differences in strain virulence may also account for the discrepant results obtained in our study compared to previous work.

We searched for loci influencing risk for symptomatic WNV disease by comparing symptomatic WNV cases to asymptomatic, WNV-positive individuals. This approach likely is more robust than using random blood donors with unknown WNV exposure history or WNV-negative individuals as controls. Groups of RBDs with an unknown WNV exposure are likely to include previously uninfected individuals with the potential to develop either WNF/M or WNE/P, and therefore may reduce the power to detect a difference between groups. By considering only asymptomatic, WNV-positive blood donors, the probability of including individuals who might develop WNF/M or WNE/P was eliminated. This strategy could explain the observation that the significant associations between *IRF3* or *MX1* and WNV disease were not replicated when RBD of unknown WNV infection status were used for comparison. Similarly, using WNV-negative controls rather than WNV-positive asymptomatic controls could confound risk factors for infection and symptomatic disease. This could explain, in part, our ability to replicate the association between *CCR5Δ32* and symptomatic infection reported by Glass *et al.* when we used sero-negative controls but not when WNV-positive asymptomatic blood donors were used as a control group [13].

The composition of the asymptomatic control cohort also appeared to influence the power to detect variants associated with risk for WNE/P. For example, inclusion of RBD in the control group with WNF/M or WNF/M and asymptomatic infection diminished the ORs and the level of significance of the association between *OAS1 rs*34137742 and WNE/P. As a corollary, since some of the asymptomatic individuals may have had mild symptoms [28], comparing both asymptomatic WNV-positive cases combined with WNF/M would mitigate errors introduced by the misclassification of asymptomatic cases.

Age is a known independent risk factor for WNV disease progression with elderly patients at the highest risk for encephalitis and death [7], [8], [29], [30], [31]. Ages were not available for many of the asymptomatic WNV-positive blood donors (233 out of 331) so that we were unable to control for age as a covariate. To test whether the analyses of WNV disease progression were robust to confounding by age, the effect of age was tested by comparing the results of a logistic regression using the subset of cases for which age information was available to those obtained for the entire set of samples. Age was available for 94% of WNE/P cases, 96% of WNF/M cases, and 30% of asymptomatic cases. The results were virtually the same, suggesting that age is not a major confounder of our analyses. Furthermore, in order for age to confound the analysis, it would have to be associated with the SNPs tested here. This could happen via settlement of different ethnic groups over the relevant time period in the regions from which cases and controls were collected. However, adjusting for ethnicity eliminated this potential issue.

In summary, we have identified two novel loci, *IRF3* and *MX1*, associated with risk of WNV disease, and a SNP in *OAS1* associated with WNE/P. Each of the SNPs responsible for the observed association signal is located in an intron and none is in strong linkage disequilibrium with known functional variants in their respective genes. Each of these loci is involved in either an interferon regulatory pathway or is an effector of the interferon response. Accordingly, these findings provide further evidence, albeit indirect, that the interferon pathway MAY play an important role in modulating human WNV disease, influencing both the risk of symptomatic infection and disease progression.

## Methods

### Study Participants

Samples from symptomatic WNV-positive patients (n = 422) were collected from the Southeast, Midwest, Northeast, Southwest, Intermountain West, and West coast regions of the United States. These cases represented individuals who sought medical attention, had signs consistent with WNV disease (e.g., fever, meningitis, encephalitis), and who were subsequently confirmed by serological testing to have been infected with WNV. Data for case classification were collected by review of medical records and in some cases interviews with patients. A WNF/M case (n = 280) was defined as an individual who had weakness, headache, acute fever, stiff neck, rash, and/or cerebrospinal fluid (CSF) pleocytosis consistent with meningitis. A WNE/P case (n = 140) was defined as an individual who experienced encephalitis or paralysis. Encephalitis was defined as fever, headache, and altered mental status ranging from confusion to coma. There were no recorded deaths among cases in our cohort. For two symptomatic cases, the phenotypic data were incomplete and further classification was not possible. WNV nucleic acid positive blood samples (n = 331) were collected in the Northeast, Midwest, Southwest, Intermountain West, and West coast regions of the United States and in Canada by Blood Systems and by a private blood bank in Colorado. Fifty-seven percent of these individuals self-reported no symptoms and 16 percent reported only one symptom (i.e. headache, body aches, or skin rash) in the week prior to donation [28]. However, only the odds of reporting skin rash were significantly higher for confirmed WNV-positive blood donors compared to uninfected donors.

All subjects and/or their legal guardians provided written informed consent and the study was approved by the Institutional Review Boards at Seattle Children's Hospital, the University of Washington, the University of Utah, Georgia State University, the University of Texas Health Sciences Center at Houston, Blood Systems, the Centers for Disease Control and Prevention, and the University of Massachusetts Medical School.

### DNA Isolation and Genotyping

DNA was isolated from 1–20 ml venous blood or white blood cells using Qiagen's (Valencia, CA) Puregene DNA purification system according to the manufacturer's instructions. Candidate SNPs, both functional and/or haplotype tagging in Caucasians, were selected from 86 genes that have been associated with WNV infections in an animal model or cell culture. In total, 384 SNPs were genotyped using an Illumina custom oligo pool assay (OPA) (Table S1). Twenty-four SNPs failed initial quality control filters and were removed from all downstream analysis. Association analyses were performed on 360 SNPs distributed across 86 genes. Of these SNPs, three deviated from Hardy-Weinberg equilibrium (HWE), but were not significantly associated with WNV infection or disease progression. A sex check was performed for all patients by comparing the reported sex in the phenotypic database to the genotypic sex. Genotypic sex was determined from the heterozygosity estimates of the X chromosome SNPs genotyped using the Illumina custom OPA. Discrepant sex values existed for seven study participants, and they were excluded from the analysis.

*CCR5Δ32* was typed manually in our cohort of symptomatic (n = 394) and asymptomatic WNV-positive cases (n = 342) using primers flanking the 32-bp deletion. Fragments were resolved on a 2% agarose gel and visualized with ethidium bromide staining. Of the 736 samples genotyped for *CCR5Δ32*, six samples did not produce reliable genotypes and seven samples failed a sex check performed using the Illumina OPA data. An additional 10 samples lacked the genotype data needed to control for population stratification in the logistic regression. After removal of these 23 samples, 713 and 712 samples were included in the association analysis for symptomatic WNV infection and WNE/P, respectively. For a single sample, phenotypic information on disease severity was not available. Therefore, this case was omitted from the analysis of disease progression. The analysis of symptomatic WNV disease compared 385 WNV symptomatic cases to 328 asymptomatic WNV-positive controls. The analysis of WNE/P compared 131 WNE/P cases to controls consisting of 581 WNF/M cases combined with asymptomatic WNV-positive blood donors.

To assess variation in the *5′ cis*-regulatory region of *CCR5*, a 1.2 kb fragment was PCR-amplified using a pair of primers that captured exons 1 and 2 and ∼1 kb of sequence upstream and downstream of each. DNA sequencing was performed using internal primers on an ABI 3130 automated sequencer. Sequence trace files were aligned and genotype calls were made using Codon Code (Dedham, MA). Polymorphic sites were confirmed by visual inspection of the traces. All SNPs were in HWE (p≥0.05).

### Association Analysis

To test whether variants were associated with symptomatic WNV infection, WNE/P and WNF/M cases were compared to asymptomatic, WNV-positive blood donors. To test whether variants were associated with WNE/P, WNE/P cases were compared to WNF/M cases and asymptomatic WNV-positive blood donors. Single nucleotide polymorphism (SNP) analysis and genetic model assessment were performed using PLINK version 1.06 (http://pngu.mgh.harvard.edu/purcell/plink/) [32]. ORs and 95% CIs were calculated using both contingency tables and logistic regression. P-values were corrected for multiple tests using a Bonferroni correction for 337 (autosomal chromosome SNPs) or 360 (autosomal and X chromosome SNPs) tests. Dominant, recessive, genotypic, additive, and allelic genetic models of inheritance were considered. For each variant tested, the odds ratios (OR), p-values, and 95% confidence intervals (CIs) (unadjusted for multiple tests) for each model of inheritance are reported in Tables S2–S15 and S18, S19. For all models, we define D as the minor allele and d as the major allele following the notation of PLINK [32]. The minor allele was defined based on the allele frequencies in this dataset. ORs for the minor allele were calculated using a dominant model (DD and Dd versus dd), a recessive model (DD versus Dd and dd), and a genotypic model (DD versus Dd versus dd). The allelic model compared D versus d. The additive model tested for the additive effects of allelic dosage. SNPs on the X chromosome were included in the allelic model only. For the contingency table analysis, allele and genotype frequency differences between cases and controls were calculated a using a 2×2 or a 2×3 table that compared the number of expected and the number of observed individuals in each category. Two sided p-values were estimated by the chi-square test of significance with 1 (2×2 table) or 2 (3×2 table) degrees of freedom. P-values were corrected for multiple tests using the Bonferroni correction. In the logistic regression analysis, we controlled for population stratification by including the first five principle components (PCs) as covariates in the model. PCs were estimated from the 360 QC filtered genotypes using EIGENSTRAT [33]. P-values were estimated using the Wald z-statistic and corrected for multiple tests using the Bonferroni correction. To determine if age should be included as a covariate in the model, logistic regression was performed on the subset of subjects with reported age both with and without age included as a covariate in the model. Additionally, we added an age*SNP interaction effect and an age*age∧2 as age effects are often non-linear. Age, age*SNP, and age*age∧2 were not found to be a significant covariates in our analysis and were left out of the logistic regression analysis. Age data were unavailable for 70% of WNV-positive blood donors, 6% of WNE/P cases, and 4% of WNF/M cases.

## Supporting Information

Table S1 **SNPs included on the Illumina Custom OPA.**
(XLS)

Table S2 **Symptomatic vs. asymptomatic genotypic contingency table analysis.**
(XLS)

Table S3 **Symptomatic vs. asymptomatic contingency table analysis using a dominant model of inheritance.**
(XLS)

Table S4 **Symptomatic vs. asymptomatic contingency table analysis using a recessive model of inheritance.**
(XLS)

Table S5 **Symptomatic vs. asymptomatic contingency table analysis using an additive model of inheritance.**
(XLS)

Table S6 **Symptomatic vs. asymptomatic logistic regression analysis results for an additive model of inheritance.**
(XLS)

Table S7 **Symptomatic vs. asymptomatic logistic regression analysis results for dominant model of inheritance.**
(XLS)

Table S8 **Symptomatic vs. asymptomatic logistic regression analysis results for a recessive model of inheritance.**
(XLS)

Table S9 **WNE/P vs. WNF/M genotypic contingency table analysis.**
(XLS)

Table S10 **WNE/P vs. WWF/M contingency table analysis using a dominant model of inheritance.**
(XLS)

Table S11 **WNE/P vs. WNF/M contingency table analysis using a recessive model of inheritance.**
(XLS)

Table S12 **WNE/P vs. WNF/M contingency table analysis using an additive model of inheritance.**
(XLS)

Table S13 **WNE/P vs. WNF/M logistic regression analysis results for an additive model of inheritance.**
(XLS)

Table S14 **WNE/P vs. WNF/M logistic regression analysis results for a dominant model of inheritance.**
(XLS)

Table S15 **WNE/P vs. WNF/M logistic regression analysis results for a recessive model of inheritance.**
(XLS)

Table S16 **CCR5 logistic regression results.**
(XLS)

Table S17 **Contingency table analysis of variants previously association with WNV disease.**
(XLS)

Table S18 **Symptomatic vs. asymptomatic allelic contingency table analysis.**
(XLS)

Table S19 **WNE/P vs. WNF/M allelic contingency table analysis.**
(XLS)

Table S20 **OAS1 SNP contingency table analysis using different meningitis classifications.**
(XLS)
